# Middle-Range Theory of Ineffective Breathing Pattern in children with Congenital Heart Disease

**DOI:** 10.1590/1518-8345.5826.3783

**Published:** 2023-01-06

**Authors:** Nayana Maria Gomes de Souza, Viviane Martins da Silva, Marcos Venícios Oliveira Lopes

**Affiliations:** 1 Universidade Federal do Ceará, Fortaleza, CE, Brazil.

**Keywords:** Nursing Diagnosis, Nursing Research, Nursing Theory, Nursing Process, Validation Study, Heart Diseases, Congenital, Diagnóstico de Enfermagem, Pesquisa em Enfermagem, Teoria de Enfermagem, Processo de Enfermagem, Estudo de Validação, Cardiopatias Congênitas, Diagnóstico de Enfermería, Investigación en Enfermería, Teoría de Enfermería, Proceso de Enfermería, Estudio de Validación, Cardiopatías Congénitas

## Abstract

**Objective:**

to develop and evaluate a middle-range theory for the Nursing Diagnosis of Ineffective Breathing Pattern in children with congenital heart disease.

**Method:**

a methodological study carried out in two stages: 1) development of a middle- range theory for Ineffective Breathing Pattern in children with congenital heart diseases from the analysis of the NANDA-International taxonomy, Callista Roy’s Adaptation Model and a literature review; and 2) assessment of the middle-range theory developed using expert panel evaluation.

**Results:**

after three panel evaluations, the final version of the middle-range theory resulted in four metaparadigms, two key concepts, two pictorial diagrams, two propositions and a description of the interrelationships between the key concepts of Ineffective Breathing Pattern in children with congenital heart diseases and evidence for the Nursing practice.

**Conclusion:**

the middle-range theory developed and evaluated by experts identified stimuli and behaviors that can assist nurses in identifying the reasons why Ineffective Breathing Pattern is diagnosed and how it manifests itself in children with congenital heart disease, increasing understanding of the relationships between the causes and their temporality.

## Resumo

**Objetivo:**

desenvolver e avaliar uma teoria de médio alcance para o Diagnóstico de Enfermagem de Padrão Respiratório Ineficaz em crianças com cardiopatia congênita.

**Método:**

um estudo metodológico realizado em duas etapas: 1) desenvolvimento de uma teoria de médio alcance para padrão respiratório ineficaz em crianças com doenças cardíacas congênitas a partir da análise da taxonomia NANDA-Internacional, modelo de adaptação de Callista Roy e uma revisão da literatura; e 2) avaliação da teoria de médio alcance desenvolvida por meio da avaliação de painéis de especialistas.

**Resultados:**

após três avaliações de painéis, a versão final da teoria de médio alcance resultou em quatro metaparadigmas, dois conceitos-chave, dois diagramas pictóricos, duas propostas e uma descrição das inter-relações entre os conceitos-chave do Padrão Respiratório Ineficaz em crianças com cardiopatias congênitas e evidências para a prática de Enfermagem.

**Conclusão:**

a teoria do médio alcance desenvolvida e avaliada por especialistas identificou estímulos e comportamentos que podem auxiliar os enfermeiros na identificação das razões pelas quais o Padrão Respiratório Ineficaz é diagnosticado e como ele se manifesta em crianças com cardiopatia congênita, aumentando a compreensão das relações entre as causas e sua temporalidade.

## Resumen

**Objetivo:**

desarrollar y evaluar una teoría de mediano alcance para el Diagnóstico de Enfermería “Patrón de Respiración Ineficiente” en niños con cardiopatías congénitas.

**Método:**

estudio metodológico desarrollado en dos etapas: 1) Desarrollo de una teoría de mediano alcance para el Patrón de Respiración Ineficiente en niños con cardiopatías congénitas a partir del análisis de la taxonomía NANDA-Internacional, del Modelo de Adaptación de Callista Roy y de una revisión de la literatura; y 2) Evaluación de la teoría de mediano alcance desarrollada por medio de una evaluación de un panel de expertos.

**Resultados:**

después de tres evaluaciones a cargo del panel, la versión final de la teoría de mediano alcance derivó en cuatro metaparadigmas, dos conceptos clave, dos pictogramas, dos propuestas y una descripción de las interrelaciones entre los conceptos clave del Patrón de Respiración Ineficiente en niños con cardiopatías congénitas y evidencia para la práctica de Enfermería.

**Conclusión:**

la teoría de mediano alcance desarrollada y evaluada por expertos identificó estímulos y comportamientos que pueden ayudar a los enfermeros a identificar los motivos por los cuales se diagnostica el Patrón de Respiración Ineficiente y cómo se manifiesta en niños con cardiopatías congénitas, mejorando así la comprensión de las relaciones entre las causas y su temporalidad.

**Highlight**

(1) The MRT more closely resembles the clinical practice.

(2) It can serve as a guiding framework for the implementation of actions to resolve IBP.

(3) It enables confirmation or exclusion of the probability of IBP diagnoses.

## Introduction

In healthy individuals there is a close relationship between the functions of the cardiovascular and respiratory systems, such that changes in the body’s metabolic needs are quickly accompanied by changes in both cardiac output and ventilation. However, in the presence of Congenital Heart Disease (CHD), the balance in this relationship is almost always disrupted^1^.

In CHD, the heart’s ability to increase systemic and/or pulmonary blood flow is limited, affecting aire availability to the alveoli and compromising ventilation through various mechanisms, with the possibility of oxygen delivery not meeting the tissues’ needs, contributing to the emergence of signs and symptoms of respiratory Nursing diagnoses^2^. An Ineffective Breathing Pattern (IBP) is an example, as it is directly associated with the ventilation process.

Thus, it is important that nurses are not only able to recognize the conditions that affect ventilation but also, above all, to understand the mechanisms that lead to this condition^3^. Thus, assessing clinical signs and symptoms and the reasons behind the Nursing diagnosis is essential to make accurate diagnoses, direct planning of the Nursing actions and quickly reverse this condition to prevent other respiratory problems.

The development of a Middle-Range Theory (MRT) for a specific Nursing diagnosis can contribute to describe, explain and predict this phenomenon in a given population, making diagnostic inferences more reliable and representative, as well as it can help care professionals develop clinical reasoning based on time logic and interactions between components of the diagnosis^4^.

Unlike ground theories, which are made up of relatively abstract concepts that are not operationally defined and attempt to explain or describe very broad aspects of human experience and response, an MRT has more concrete and specific concepts and propositions restricted to the real world and can be empirically tested^5^.

There is a growing body of research that evaluates accuracy of the IBP diagnosis characteristics^2^ ^,^ ^6^. Although comprehensive, these research studies do not offer insights into the reasons that lead to the development of this Nursing diagnosis and are not derived from a ground Nursing theory.

Therefore, due to the absence of more robust studies, that is, which consider all the components of the Nursing diagnoses and how they interact for the occurrence of the diagnosis, it was decided to develop an MRT on the IBP diagnosis based on Roy’s Adaptation Model with the aim of contributing to supporting diagnostic reasoning, thus subsidizing better quality Nursing care for children with congenital heart disease.

As it is believed that children with congenital heart diseases constantly seek to adapt their breathing pattern to their body’s needs, Roy’s Adaptation Model was chosen as a conceptual model to support this MRT^7^. This established Nursing theory uses concepts inherent to the individual’s adaptive process and involves different coping mechanisms. One of these mechanisms is focused on human physiology, in which the oxygenation component is found. In this component, Roy lists concepts that are classified as stimuli, that is, those that provoke a human response, and as behaviors, which refer to the individual’s manifestations regarding stimuli^7^.

The objective of this study was to develop and evaluate the components of an MRT that defines and explains the elements and processes that lead to establishing the Nursing diagnosis of IBP in children with CHD based on the NANDA-International (NANDA-I) taxonomy, Roy’s Adaptation Model and a literature review.

## Method

### Study design

This is a theoretical and a methodological study carried out in two stages: 1) development of an MRT for the Nursing diagnosis of IBP in children with CHD; and 2) evaluation of the MRT developed by a panel of experts.

### Stage 1 - Development of an MRT for the Nursing diagnosis of IBP in children with CHD

This MRT was developed in 5 phases: definition of the approach to construct the MRT, definition of key concepts, development of pictorial diagrams, elaboration of propositions and establishment of causal relationships and evidence for the clinical practice^4^.

#### Definition of the approach to construct the MRT

The initial step in the development of this theory was to analyze and synthesize theoretical and empirical knowledge about the phenomenon of interest (IBP). Construction of the MRT for the Nursing diagnosis of IBP in children with CHD was based on concepts related to the physiological oxygenation mode of Roy’s Adaptation Model, an integrative literature review and diagnostic elements of IBP using the NANDA-I taxonomy.

The integrative literature review was carried out to select the concepts and studies included published until the first half of 2020. The guiding question was as follows: “Which elements characterize and are associated with the manifestation of the Nursing diagnosis of Ineffective Breathing Pattern in children with congenital heart diseases that do not undergo full correction?” From this, the “pulmonary ventilation”, “breathing” and “congenital heart disease” descriptors, associated through the Boolean operator “AND”, were used to search the PubMed, CINAHL, Web of Science and Scopus databases.

The following filters were used in the research: publications available in full text between 2006 and 2020 in English, Portuguese and Spanish. Experimental, validation and review studies were selected, excluding editorials and letters to the editor. The initial sample consisted in 392 articles from PubMed, 1,494 from Scopus, 180 from Web of Science and 21 from CINAHL. After reading the titles and abstracts, the sample was reduced to 25 from PubMed, 33 from Scopus, 17 from Web of Science and 11 from CINAHL. Finally, 55 articles were selected: 14 from PubMed, 20 from Scopus, 13 from Web of Science and 8 from CINAHL.

#### Definition of key concepts

From the analysis of Roy’s Adaptation Model, NANDA-I and the literature review, key concepts related to the phenomenon of interest (IBP) were selected. Thus, we grouped the concepts according to Roy’s Adaptation Model classification into stimuli, reflecting the clinical factors before diagnosis, and into behaviors, representing the clinical factors resulting from IBP in children with CHD.

The related factors, at-risk population and associated conditions of the NANDA-I taxonomy, in addition to the etiological factors listed in the literature, are classified as stimuli. Likewise, the defining characteristics of NANDA-I and the clinical indicators of IBP in children with CHD found in the literature are classified as behaviors.

The stimuli listed for this MRT were classified as focal and contextual according to the degree of influence they exert on the population studied, using the nomenclature proposed in Roy’s Adaptation Model. Focal stimuli include factors that are internal or external to the individual and which exert direct impacts on the person^7^. They were subdivided into precipitating agents, those which are a direct cause of the diagnosis, and predisposing agents, those which increase an individual’s susceptibility to a given condition^4^.

Contextual stimuli include those that enhance stimuli linked to the individual^7^, which can be considered disabling or reinforcing agents, amplifying the effects of other stimuli. Disabling agents affect the recovery period or may hinder adoption of health-promoting behaviors, resulting in health problems or extending the effect of other causative agents. Finally, reinforcement agents act specifically as enhancers of pre-existing conditions^4^.

The behaviors were divided into acute and chronic. Acute behaviors include signs and symptoms that represent the initial spectrum of the presence of inadequate ventilation in children with CHD; they were subdivided into confirmatory signs (those with high specificity measures that can help nurses confirm the diagnosis) and signs detectable in pulmonary function tests and clinical deterioration (when there is exacerbation of the respiratory condition resulting from the changes inherent to decompensated heart disease) according to the clinical course of the diagnostic inference. On the other hand, chronic behaviors correspond to clinical manifestations of IBP that occur progressively over months or years.

#### Development of pictorial diagrams

After analyzing the results, two pictorial diagrams were developed, one for the stimuli and the other for the behaviors, with the purpose of visualizing the connections between the stimuli and the phenomenon under study and the behaviors produced by the children with CHD, respectively.

We adopted the Ishikawa diagram model (cause-effect) to represent the stimuli and used a decision tree diagram for the behaviors.

#### Elaboration of propositions

Two propositions were formulated according to a critical analysis of the pictorial diagrams to highlight the existing relationships of the stimuli and behaviors with the study phenomenon. These propositions represent hypotheses of the relationships between the concepts of the theory to be tested empirically^5^.

#### Establishment of causal relationships and evidence for the clinical practice

In the last stage, the interrelationships between stimuli and behaviors related to IBP in children with CHD were established and described, providing a better understanding of the clinical use of the Nursing diagnosis^4^. To better understand these relationships, examples were described for each causal event.

### Stage 2 - Expert panel evaluation of the MRT developed

After development of the MRT for IBP in children with CHD, its assessment was performed using panel evaluation to verify with experts whether the MRT for the IBP Nursing diagnosis represents the diagnostic construct in the actual clinical practice among the pediatric population with CHD.

### Participants and study variables

The experts were divided into two groups. The first group included nurses with at least 5 years of clinical practice with children with CHD. For the second group, the following criteria were adopted: nurses who had at least a master’s degree and publications on Nursing diagnoses according to the NANDA-I taxonomy and/or Nursing theory.

The experts were selected according to their curricula in the Lattes Platform and the sampling strategy used was of the “snowball” type. Recruitment, by means of email invitations, included communication of the study justifications, objectives, evaluation form and deadline for response. After accepting and signing the Informed Consent Form, the participants entered the data collection phase for evaluation.

In the first panel evaluation, the experts were asked about the relevance of each of the concepts identified in the review. Initially, a list of concepts was submitted to the judges with 10 stimuli and 21 behaviors, and of these, nine stimuli and all behaviors were considered relevant. The stimulus that did not show statistical significance was age less than two years old; therefore, it was excluded^8^.

For the second panel evaluation, all the MRT components (metaparadigms, concepts, pictorial diagrams, propositions, and causal relationships) were sent to the experts for evaluation. In this panel, the experts were asked to analyze the MRT components based on the criteria for evaluating Nursing theories proposed by Fawcett: significance, internal consistency, parsimony and pragmatic adequacy of the components of the MRT developed^5^.

Significance refers to the theory’s justification capacity for the Nursing discipline. Internal consistency concerns the theory concepts’ ability to present semantic clarity (when a constitutive definition is given for each concept), semantic consistency (when the same term and definition are used for each concept in every theory) and structural consistency (when the concepts used in the theory are interrelated with no contradictions in the relational propositions). Parsimony refers to the theory’s ability to economically apply concepts and statements to explain the phenomenon of interest and, finally, pragmatic adequacy concerns the theory’s ability to recommend socially significant actions that lead to favorable results^5^.

### Period and data collection

The assessments regarding of all the MRT components were based on a Likert scale, with several variable levels, with 1 and 5 being the lowest and highest agreement levels, respectively. In the case of negative answers, the components underwent changes according to the judges’ suggestions. Of the 23 experts from the first panel evaluation, only 20 (11 with academic experience in the use of Nursing diagnoses and/or Nursing theories and 9 with clinical experience) agreed to participate in the study and returned the completed data collection instrument within 90 days. This data collection period was from September to November 2020.

The components were changed with feedback from the second panel evaluation and then submitted for a third evaluation round aiming at defining the final version of the MRT. This data collection period took place in December 2020.

### Data treatment and analysis

To evaluate the MRT for IBP in children with CHD, the Content Validity Index (CVI) was calculated in all three panel evaluations. We decided to use the weighted median as the CVI estimate due to identifying non-normality in the distribution of the estimates. In addition to the estimate of the CVI median, 95% confidence intervals were also calculated for each median, as well as the Wilcoxon test for weighted median, with a CVI greater than or equal to 0.9. Thus, the component would be considered valid for significance, internal consistency, parsimony and pragmatic adequacy if the descriptive level (p-value) found in the Wilcoxon test was greater than 0.05.

### Ethical aspects

All ethical and legal requirements for research with human beings were met, with approval by the Research Ethics Committee, under research approval certificate number 2,253,626.

## Results

The data presented in Table 1 show the assessment in the second panel evaluation regarding the questions pertinent to the criteria for evaluation of Nursing theories as specified by Fawcett. All components reached the desired levels regarding significance, parsimony and pragmatic adequacy.

The items that did not reach CVI ≥ 0.9 are related to structural consistency, referring to internal consistency of the theory. Thus, for some experts, there were inconsistencies between the description of causal relationships and both pictorial diagrams. These components underwent changes as suggested by the panel and were resubmitted for consideration in the third round.

Thus, the final version of the components of the theory developed resulted in four metaparadigms, two key concepts, two pictorial diagrams, two propositions and a description of the interrelationships between the IBP key concepts in the children with CHD, with positive feedback for all criteria on the evaluation of Nursing theories specified by Fawcett. All the components modified in the third panel evaluation reached a CVI value of 1.00.

**Table 1:** Experts’ assessment of Fawcett’s criteria for the evaluation of Nursing theories (second panel evaluation round)

Fawcett’s Criteria for the Evaluation of Nursing Theories and Pertinent Questions	p-value*	CVI^†^	95% CI^‡^		*p-*value^§^
**1. Significance**					
The metaparadigms are explicit	<0.001	1.00	1.00	1.00	0.997
Influential Nursing authors are explicit or cited in the bibliography	<0.001	1.00	1.00	1.00	1.000
The conceptual model from which the theory was derived is explicit	<0.001	1.00	1.00	1.00	0.975
The philosophical claims on which the theory is based are explicit	<0.001	1.00	1.00	1.00	0.975
**2. Internal consistency**					
The concepts present semantic clarity	<0.001	1.00	1.00	1.00	1.000
The concepts present semantic coherence	<0.001	1.00	1.00	1.00	0.997
The propositions present structural consistency	<0.001	1.00	0.88	1.00	0.729
The causal relationships present structural consistency	<0.001	0.88	0.87	1.00	0.300
The pictorial diagrams present structural consistency	<0.001	0.88	0.87	1.00	0.300
The context (philosophical claims and conceptual model) and content (concepts and propositions) of the theory are congruent	<0.001	1.00	1.00	1.00	0.997
**3. Parsimony**					
The theory content is presented clearly and concisely	<0.001	1.00	1.00	1.00	0.997
The concepts are presented clearly and concisely	<0.001	1.00	1.00	1.00	0.997
The propositions are presented clearly and concisely	<0.001	1.00	0.88	1.00	0.895
**4. Pragmatic adequacy**					
The theory is applicable to the Nursing practice context	<0.001	1.00	1.00	1.00	1.000
It is feasible to implement practices derived from the Theory	<0.001	1.00	1.00	1.00	0.997
Nurses have the legal capacity to measure the accuracy of Nursing diagnosis based on the Theory	<0.001	1.00	1.00	1.00	0.997

*Shapiro-Wilk test; ^†^Content Validity Index; ^‡^95% Confidence intervals; ^§^Wilcoxon test - Values that did not show variability

### A Middle-Range Theory for the Nursing diagnosis of Ineffective Breathing Pattern in children with congenital heart disease

#### Metaparadigms

In the context of this MRT, the person is represented by a child with CHD being holistically cared for to promote adaptation. The environment is represented by all circumstances and conditions that negatively or positively affect ventilation of the child with CHD. Health includes ventilatory integrity maintenance in children with CHD. Finally, the Nursing goal is to obtain adaptive responses for efficient ventilation that will contribute to maintaining integrity of the child with CHD.

#### Key concepts

The stimuli are the elements that cause the response; they can negatively influence the ventilation process and corroborate installation of the IBP diagnosis in children with CHD. The behaviors can be observed when the ventilation process does not occur satisfactorily; that is, they are consequent adaptive reactions to IBP stimuli.

**Figure 1:**
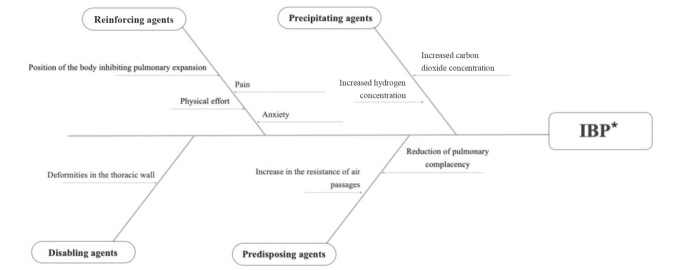
Pictorial diagram representing the causal relationships between stimuli of Ineffective Breathing Pattern in children with congenital heart diseases

Figure 1 shows all stimuli listed in the first panel evaluation and illustrates the causal relationships between the stimuli of IBP in children with CHD, highlighting the hierarchical and time-based structure of the relationships between the stimuli, in which the contextual elements (disabling and reinforcing agents) influence the focal elements (predisposing and precipitating agents) in the occurrence of IBP.

*IBP = Ineffective Breathing Pattern

**Figure 2:**
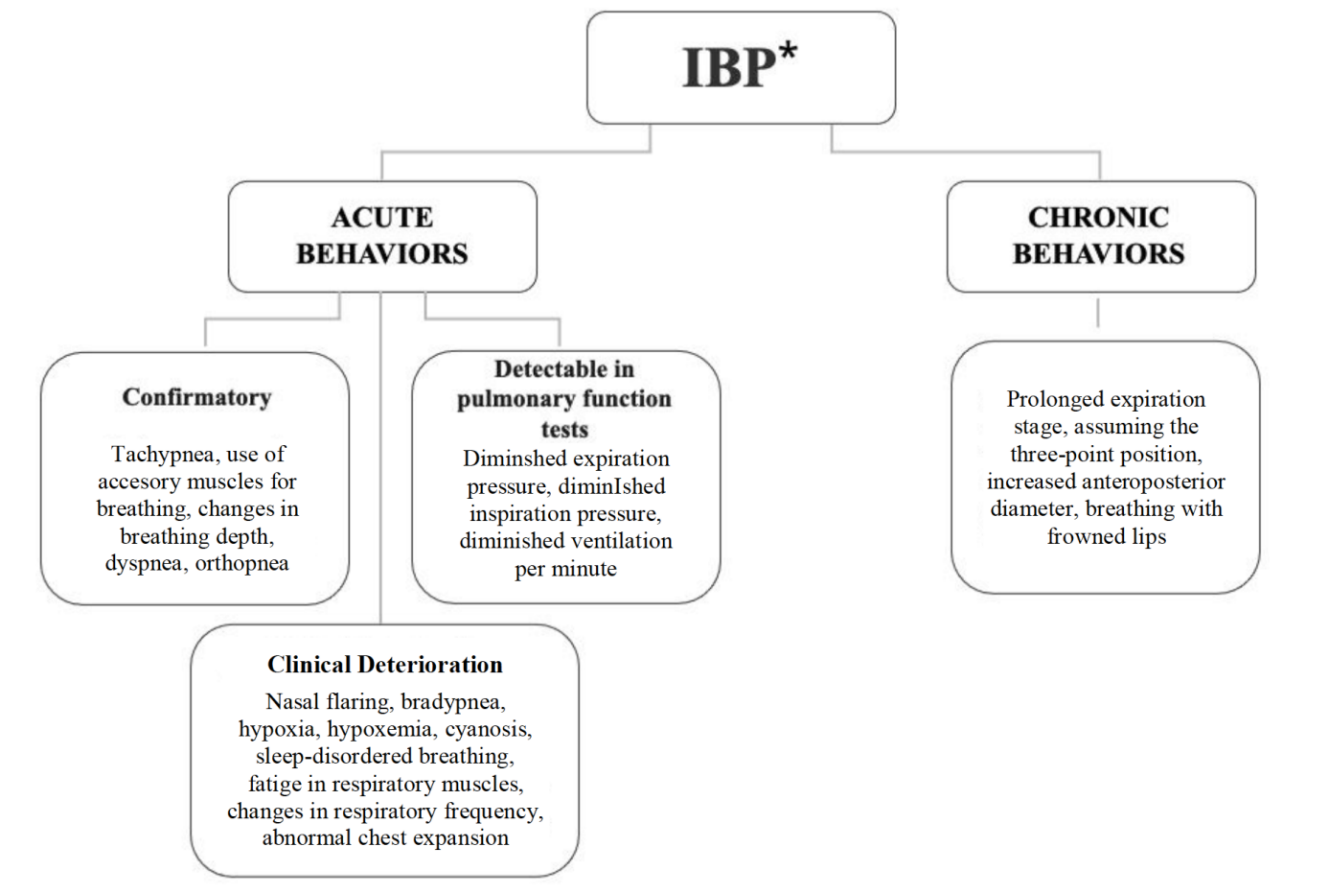
Pictorial diagram representing the diagnostic inference of the behaviors of Ineffective Breathing Pattern in children with congenital heart diseases

To illustrate and present the behaviors of IBP in children with CHD evaluated in the first panel evaluation, a pictorial diagram was created in a structure similar to a tree, in which the behaviors were subdivided into acute or chronic branches and the leaves represented the set of signs and symptoms during diagnostic inference (Figure 2).

*IBP = Ineffective Breathing Pattern

It is noted that the list of stimuli and behaviors obtained for this MRT were conceptually and operationally defined to verify the existing relationships between them as a result of the clinical and critical reasoning process.

#### Propositions

The following propositions were elaborated based on the previously established concepts of stimuli and adaptive responses:

1. Contextual stimuli can enhance the effect of focal stimuli, which in turn exert direct influences on IBP emergence.
2. Acute confirmatory behaviors and those detectable in pulmonary function tests support or refute the IBP diagnosis, whereas acute behaviors related to clinical deterioration may be present when there is exacerbation of the respiratory condition, and chronic behaviors appear after months or years of ventilatory changes resulting from CHD.

## Discussion

### Establishment of causal relationships and evidence for the clinical practice

Focal stimuli increase resistance of air passages and reduce pulmonary complacency; they act as predisposing agents by increasing the susceptibility of children with CHD to IBP^9^.

There are many clinical conditions in children with CHD that hinder passage of air, thus causing an increase in the resistance of air passages and compromising ventilation; some of these include abnormal anatomical structures (enlarged pulmonary artery, enlarged left atrium and massive cardiomegaly), pulmonary hypertension, increased pulmonary blood flow and respiratory infections. The clinical conditions that contribute to hampering lung expansion and thus to reducing pulmonary complacency are presence of intra-alveolar and interstitial fluid, congestive heart failure, pulmonary congestion, and increased pulmonary blood flow^8^ ^-^ ^9^.

The focal stimulus increases the carbon dioxide and hydrogen concentrations, acting as precipitation agents; that is, those that initially trigger the study phenomenon and cause an imbalance in the ventilation-perfusion process, triggering behaviors associated with the IBP Nursing diagnosis in order to compensate or correct the change in these values^8^ ^,^ ^10^ ^-^ ^11^.

Although it cannot be considered a clinical condition, physical effort is a contextual stimulus that influences ventilation alteration in children with CHD, as the activity requires an increase in respiratory work, which requires more oxygen in the respiratory muscles, thus causing a reduction in muscle strength. This can result in respiratory distress due to limited oxygen supply in children with CHD^12^.

Respiratory changes in children with CHD may also be associated with contextual stimuli anxiety^13^. When this type of situation occurs, the brain’s perception of suffocation erroneously signals shortness of breath, inappropriately activating the alarm system. Misinterpretation of these symptoms increases fear and activates the autonomic nervous system, thereby causing tachypnea to reverse the increase in carbon dioxide concentration^13^.

Pain is also a contextual stimulus that contributes to the emergence of a breathing pattern inconsistent with the metabolic needs of a child with CHD because it activates the sensory system involved in breathing and thus impairs performance of the muscles involved in lung expansion^3^.

The position of the body is another contextual stimulus that reinforces the emergence of IBP, as some body positions may restrict respiratory movements and inhibit pulmonary expansion^14^. Deformities in the thoracic wall are a contextual stimulus that can restrict mobility of the rib cage and/or spine, resulting in a decrease in the efficiency of the respiratory muscles and a consequent reduction in expandability and static pulmonary volumes^15^.

The first four contextual stimuli cited act in causality processes as reinforcing agents, and the last stimulus acts as a disabling agent^3^ ^,^ ^12^ ^-^ ^15^.

Confluence of the stimuli described leads to the establishment of a series of behaviors that characterize the Nursing diagnosis of IBP in children with CHD. In acute behaviors, the rapid deterioration of respiratory function leads to the emergence of more intense clinical manifestations, representing more direct evidence of the diagnosis in question.

Confirmatory acute behaviors include tachypnea, dyspnea and alterations in breath depth in children with CHD to reduce the excess hydrogen and carbon dioxide in the blood that reaches the lungs, thus acting as a compensatory mechanism, appearing as an attempt to compensate for the imbalance between oxygen supply and demand^2^.

On the other hand, the confirmatory acute behavior of using accessory muscles for breathing arises from the need to generate greater efforts of the respiratory muscles to overcome airway resistance, therefore satisfying the increased ventilatory need to optimize ventilation and gas exchange^2^. Finally, orthopnea, the confirmatory acute behavior, is attributed to the air diffusion limitation caused by an increase in the resistance of air passages and reduced pulmonary complacency, exacerbating respiratory distress and preventing the patient from remaining in the supine position^2^.

There are acute behaviors that depend on the performance of pulmonary function tests for the evaluation. Carrying out these tests makes it possible to quantify and monitor the strength of the respiratory muscles. For example, the decrease in pressure generated in the mouth after a complete inspiration or expiration, that is, diminished inspiratory or expiratory pressure, respectively, reflects the increase in the resistance of air passages^16^.

The decrease in the volume of air moved into the respiratory tract every minute can be caused by reduced tidal volumes, which occur in restrictive conditions, such as reduced pulmonary complacency that limits chest expansion, common in children with CHD^16^. Therefore, the ventilation *per* minute value can be used to assess ventilation effectiveness.

Hypoxemia and cyanosis are acute behaviors of clinical deterioration that occur when there is no effective gas exchange at the alveolar level, causing a decrease in oxygenation in the arterial blood^8^ ^,^ ^17^ ^-^ ^18^. On the other hand, the acute behaviors of clinical deterioration hypoxia and fatigue of the respiratory muscles are the result of insufficient oxygen supply for a given metabolic rate, causing anaerobic metabolism, that is, an increase in the lactate rate and a decrease in serum bicarbonate for hypoxia and incoordination of respiratory movements for fatigue of the respiratory muscles^8^ ^,^ ^18^.

The change in the respiratory rhythm is an acute behavioral indicator of clinical deterioration that occurs as the respiratory effort is increased to compensate for the excess carbon dioxide present in the blood, thus influencing the peripheral and central chemoreceptors in periodic sequence causing irregular breathing movements^2^ ^,^ ^18^.

Nasal flaring is another acute behavior of clinical deterioration sensitive to respiratory effort and appears as an attempt by the body to reduce resistance of the upper airways^2^ ^,^ ^18^. Another clinical deteriorating behavior is sleep-disordered breathing. In these cases, during the sleep period, there is reabsorption of peripheral edema leading to systemic and pulmonary hypervolemia, with consequent worsening of pulmonary congestion that leads to a reduction in pulmonary compliance^19^.

When the child has atelectasis, respiratory infections, pleural effusion and conditions that compromise pulmonary compliance and resistance of air passages, asymmetry of the ventilatory movements between both hemithoraxes may be perceived; in this case, the child exhibits acute clinical deterioration and abnormal chest expansion^3^.

The decrease in respiratory frequency (bradypnea) signals impending respiratory arrest in children with CHD because the body is no longer able to compensate for the increased metabolic needs^2^.

In addition to the aforementioned acute behaviors, there are others which characterize phenomena that are chronic, where ventilatory changes occur progressively over months or years. CHDs can be seen as chronic diseases characterized by their long course (sometimes incurable), their imposition of limitations on the individual, and the constant demand for adaptations to the breathing pattern^20^. In these situations, the clinical manifestations may be more subtle.

The three-point position, characterized by projection of the chest forward and support of both arms parallel to the body, elevating the shoulders, contributes to improving the diaphragmatic function due to the reduction in tension in the abdominal muscles. It also allows the muscles of the upper limbs and shoulders to act more effectively as accessory muscles for breathing, thus promoting an improvement in ventilation^21^.

Breathing with pursed lips was another behavior pointed out in the literature review as a chronic behavior. This maneuver triggers adaptations, increasing arterial oxygen levels and saturation, as well as reducing the carbon dioxide rate^22^. The increase in the anteroposterior diameter of the chest occurs due to the constant need to increase lung expansion to optimize the ventilatory response^18^.

The “prolonged expiration stage” chronic behavior manifests itself when expiration becomes laborious and prolonged. In this case, expiration time increases to maintain high airway pressure^18^. It is noted that this behavior is confirmed or refuted by means of a pulmonary function test.

Finally, for the inference of IBP in this MRT, nurses will identify a set of behaviors and evaluate the relationships between these behaviors and the clinical situation in which the child with congenital heart disease is. Therefore, determination of the IBP diagnosis is performed based on the nurses’ confidence in relation to accuracy of the behaviors^2^ ^,^ ^6^ ^,^ ^23^.

After defining the diagnostic “status”, nurses will identify a set of stimuli that can lead to IBP development and the data analysis will include a separate description of the groups with and without the IBP diagnosis with subsequent application of statistical inference methods for its comparison, thus establishing the causal relationships of IBP development in this population^23^.

### Evaluation of the theory

Evaluation of the theory by experts with different world views, experiences, scientific knowledge and creativity allows verifying its strengths and limitations, as well as the need to add new elements to the theory or improve those that are already included in it^24^.

The first stage of this evaluation is focused on the significance that requires justification of the importance of the theory for the Nursing discipline^5^. The MRT developed was derived from Roy’s Adaptation Model, identifying the stimuli that trigger or potentiate IBP, in addition to analyzing how the behaviors of this diagnosis are manifested in the population studied. Therefore, for the experts, the significance criterion was reached because the MRT of IBP in children with CHD can contribute to scientific knowledge.

On the other hand, the internal consistency criterion requires assessing whether there is congruence between the context (conceptual model) and the content (concepts and propositions) of the theory^5^. According to the evaluation by three experts, both pictorial diagrams made it difficult to visualize a time-based structure of the relationships between the stimuli and occurrence of the study phenomenon (IBP), as well as it was not possible to visualize the spectrum (acute or chronic) of the IBP behaviors. In the causal relationships, two experts suggested that the stimuli “increased resistance of air passages” and “reduced pulmonary complacency” should be presented with the same term, in a standardized way, and not with similar terms. These components were changed as recommended by the experts and resubmitted for consideration in the third panel evaluation round.

The third criterion, parsimony, consists of evaluating the content of the theory in terms of clarity and precision, that is, the smaller the number of concepts and propositions used to clearly explain the theory, the more parsimonious the theory^5^. For the experts, the concepts, and propositions presented in this MRT satisfactorily explain the mechanisms that lead to Ineffective Breathing Pattern and how they manifest in the pediatric population with CHD.

The fourth and last criterion evaluated was pragmatic adequacy, which consists of evaluating the theory for the Nursing practice, leading to favorable results^5^. According to the experts, the concepts and propositions of the MRT can contribute to diagnostic decision-making, thus enabling nurses to intervene early in time, favoring maintenance of the breathing pattern.

This MRT makes an important contribution to furthering research in children with CHD, as well as to clinical practices, as it provides a useful tool for Nursing professionals to efficiently detect signs of developing IBP diagnosis in this population. Therefore, the results of this theory can provide the basis for moving forward with new research stages to empirically prove/study the concepts and propositions identified.

However, this research has some limitations. The evaluation has a subjective nature, being necessary to verify applicability of this MRT in the clinical practice. It was not possible to present the experts’ proficiency levels in relation to Nursing theories; in addition, the inexperience of clinical practice experts in evaluation of theories contributes to the loss of assessment by three experts from the first to the second panel evaluation rounds.

## Conclusion

Development and experts’ evaluation of this MRT regarding the IBP Nursing diagnosis identified stimuli and behaviors that can help nurses identify the reasons that lead to the diagnosis and how it manifests itself in children with CHD in the practice to improve understanding of the relationships between them and their temporality.

In this sense, the MRT more closely resembles the clinical practice, as it deals with concepts in a less abstract way and can serve as a guiding framework for implementing actions to resolve this condition, minimize the risk of developing other respiratory Nursing diagnoses and minimize discrepancies in nurses’ clinical judgment, enabling confirmation or exclusion of the probability of the diagnosis based on the current understanding of health behaviors in this population. However, further research is necessary to empirically test the concepts and propositions listed in this MRT. This theoretical framework can only be confirmed or refuted based on empirical tests.
